# Exploiting dependencies of pairwise comparison outcomes to predict patterns of gene response

**DOI:** 10.1186/1471-2105-15-S11-S2

**Published:** 2014-10-21

**Authors:** Nam S Vo, Vinhthuy Phan

**Affiliations:** 1 Department of Computer Science, The University of Memphis, Memphis, TN 38152, USA; 2 Department of Computer Science, The University of Memphis, Memphis, TN 38152, USA

**Keywords:** gene expression, small sample size, pairwise comparison

## Abstract

**Background:**

The analysis of gene expression has played an important role in medical and bioinformatics research. Although it is known that a large number of samples is needed to determine the patterns of gene expression accurately, practical designs of gene expression studies occasionally have insufficient numbers of samples, making it difficult to ascertain true response patterns of variantly expressed genes.

**Results:**

We describe an approach to cope with the challenge of predicting true orders of gene response to treatments. We show that true patterns of gene response must be *orderable *sets. In experiments with few samples, we modify the conventional pairwise comparison tests and increase the significance level *α *intelligently to deduce orderable patterns, which are most likely true orders of gene response. Additionally, motivated by the fact that a gene can be involved in multiple biological functions, our method further resamples experimental replicates and predicts multiple response patterns for each gene.

Using a gene expression data set of Sprague-Dawley rats treated with chemopreventive chemical compounds and DAVID to annotate and validate gene sets, we showed that compared to the conventional method of fixing *α*, this method increased enrichment significantly. A comparison with hierarchical clustering showed that gene clusters labelled by response patterns produced by our method were much more enriched. One of the clusters contained 3 transcription factors, which hierarchical clustering failed to place into one cluster, that have been found to participate in multiple biological networks. One of the transcription factors is known to play an important role in pathways affected by the studied chemical compounds.

**Conclusions:**

This method can be useful in designing cost-effective experiments with small sample sizes. Patterns of highly-variantly expressed genes can be predicted by varying *α *intelligently. Furthermore, clusters are labeled meaningfully with patterns that describe precisely how genes in such clusters respond to treatments.

## Background

DNA microarrays and recent high-throughput technologies such as RNA-Seq that enable the measurement of gene expressions have played an important role in medical and biological research. Consequently, there have been a great amount of work dedicated to the generation, understanding and analysis of gene expression data. This work includes statistical methods that help practitioners select differentially expressed genes appropriately, set sample size, design experiments and interpret results meaningfully; and computational methods that help clustering and classifying gene expression data.

Gene-expression studies typically aim at understanding genetic mechanisms that affect cells at different time points, drug doses, types of drugs, or any combination of these. While a majority of gene expression studies involve a small number of different types drugs, researchers have designed experiments with hundreds of chemicals at various doses and durations [1,2]. In such studies, researchers are interested in understanding not only the effects of certain drugs (compared to untreated) but also the differences and similarities among the drugs themselves.

In analyzing gene expression data that contain more than one types of drugs, one approach is to employ pairwise comparisons to determine the relative orders of genes responding to treatment pairs [3-7]. In the simplest case of having two treatment groups (e.g. treatment *t *versus untreated (control)), pairwise comparisons would identify genes that are up-regulated or down-regulated by *t*. But as the number of different types of drugs (treatments) increases, it becomes harder to interpret patterns observed from pairwise comparisons. To represent complex patterns of outcomes produced by pairwise comparisons, researchers have used ternary digits [3,5,7] and directed graphs [6]. An appropriate representation facilitates the analysis and prediction of true patterns of gene response. Furthermore, instead of making $\left(\begin{array}{c} n\\ 2 \end{array}\right)$ measurements (*n *is the number of treatments), researchers have proposed to make only Θ(*n*) measurements and use *post hoc *pairwise comparisons to derive gene response to all treatment pairs [3,8]. Pairwise comparisons of expression of genes in a network was employed to detect network perturbation [9]. The outcomes of pairwise comparisons helped the authors to study network changes to different conditions. Pairwise comparison of gene expression among selected pairs of genes can be used to create two-gene predictors with simple decision rules for classification of expression profiles [10].

One of the most challenging problems in analyzing gene expression data is that the experiment contains only few samples (or replicates). If the sample size is too small, the true pattern of highly variantly expressed genes cannot be captured accurately and consequently it is very hard for downstream analyses to predict true patterns of response. Researchers have recognized that sufficient large numbers of samples are needed to account for biological variation regardless of the underlying technologies (microarrays or RNASeq) [11-17] and even concluded that sample size should be calculated to meet the objectives of the specific aims of each study [18]. Nevertheless, due to cost, practical experimental designs tend to have small sample sizes making it hard for conventional methods to predict true response patterns of highly variantly expressed genes.

In this paper, we introduce an improvement on a previously introduced method [6] in predicting true orders of gene response to multiple treatments. We show that true patterns of response must be *orderable sets*. This property enables the exploitation of dependencies among outcomes of statistical tests that constitute patterns of gene response. Specifically, instead of obeying the convention of fixing the level of significance *α *at 0.05, we show that *α *might be varied (in a different way for each gene) to predict true patterns more accurately. In fact, by varying *α *intelligently, we could produce clusters that had more than twice the amount of functional enrichment. A comparison to hierarchical clustering showed that this method produced clusters that were much more functionally enriched.

Motivated by the fact that a gene might participate in multiple biological functions or be involved in multiple molecular processes, we show how to manipulate experimental replicates to predict and assign multiple patterns to each gene. Gene set enrichment analysis revealed that this approach of assigning multiple patterns to genes further increased functional enrichment manyfold.

## Methods

Our method consists of two main phases. First, we select significantly differentially expressed genes. Second, we assign patterns to significantly expressed genes based on their responses to all pairs of treatment groups. We will show that *orderable sets *are most likely true patterns of gene response. Based on this line of reasoning, we show how to increase the significance level *α *used in the statistical tests that determine patterns so that orderable patterns can be observed. We argue that this procedure will likely yield true patterns of gene response. Further, we describe how to use bootstrap to generate additional samples and predict most probable true patterns for each gene. This implies that each gene can bear multiple patterns and contribute to multiple enriched clusters.

### Using pairwise comparisons to determine relative orders of gene response to treatments

A pattern of a gene is defined in terms of how it responds to all treatment pairs. Before we can define how to obtain patterns for genes, we need to specify how to determine *relative orders *of gene response to treatments. Suppose that we have an experiment consisting of *K *treatment groups and each group has *R *replicates. We will suppose that the *control group *is one of the *K *treatment groups. The relative orders of gene response to treatment groups are based on pairwise comparisons.

First, we select significantly differentially expressed genes. This process can be done in a number of ways, for example, using the Kruskal-Wallis test. Additionally, the gene selection can be adjusted for false discovery rate using methods such as [19]. After significantly expressed genes have been selected, relative orders of gene responses to treatment groups are determined based on *post hoc *pairwise comparisons as follows.

Suppose that {*A*_1_*, ..., A_r_*} and {*B*_1_*, ..., B_s_*} represent the expression measurements of gene *g *under treatments *A *and *B*, respectively. The relative order of *g *responding to *A *and *B *can be determined from comparing these two groups of measurements using a pairwise-comparison test such as a two-sided Wilcoxon rank-sum test. The outcome of this test can be one of three possibilities:

**Algorithm 1 **Pattern(*S, α*)

1: Compute p-values computed in comparison tests for all treatment pairs based on samples *S*

2: Let *P *be the set of all outcomes computed based on *α*

3: **return ***P*

1 *A *≺ *B*, when *H*_0_: *µ_A _*= *µ_B _*is rejected in favor of *H*_1_: *µ_A _< µ_B_*, with p-value less than *α *(e.g. 0.05). This means *g *responds more to *B *than to *A*.

2 *B *≺ *A*, when *H*_0_: *µ_A _*= *µ_B _*is rejected in favor of *H*_1_: *µ_A _> µ_B_*, with p-value less than *α *(e.g. 0.05). This means *g *responds more to *A *than to *B*.

3 *A ~ B*, when *H*_0_: *µ_A _*= *µ_B _*is accepted. This means either there is no difference between *A *and *B*, or sample size is too small to determine the relative order.

With *K *treatment groups, there are $\frac{K\left(K-1\right)}{2}$ comparisons, resulting in $\frac{K\left(K-1\right)}{2}$ outcomes, each of which is one of the three possibilities described above. These collective outcomes constitute *the pattern of the gene g*. This procedure is summarized in Algorithm 1, which takes as input the samples of a gene responding to all treatments and a specific confidence level *α*. Note that the determination of such relative orders of treatment pairs with respect to *g *is independent of expression of other genes. Thus, the pattern of *g *is determined independently of patterns of other genes.

### Using orderable sets to predict true patterns

Let *P_g _*be the set of all $\frac{K\left(K-1\right)}{2}$ outcomes obtained from $\frac{K\left(K-1\right)}{2}$ pairwise comparisons using the procedure described in the previous section. When the pattern *P_g _*of a gene is observed at a confidence level *α*, it may not be the true pattern of response, especially if the number of samples is small.

To predict true patterns of response, we rely on the following assumptions:

*• *If a gene's responses to two treatments *A *and *B *are statistically indistinguishable (i.e. the comparison test results in *A ~ B*) *no mater how large the sample size is*, then effectively the gene responds *identically *to *A *and *B*. In this case, we say *A ≡ B*.

*• ≡ *is an equivalence relation. In other words, if *A ≡ B *and *B ≡ C*, then *A ≡ C*.

We think that these assumptions are reasonable and perhaps true for most genes and treatments. The first assumption means that if a gene responds differently to two treatments, then that difference can be detected with enough samples. The second assumption means that if we cannot differentiate between two treatments *A *and *B*, and *B *and *C*, respectively, no matter how many samples available, then we cannot differentiate *A *and *C*. It is possible that there is a paradoxical scenario under which we cannot tell the difference between *A *and *B*, and *B *and *C*, respectively, even with infinite samples, and yet we can tell the difference between *A *and *C*. We stipulate that this scenario does not occur for most genes and perhaps is non-existent. These assumptions make it possible to reason about properties of true patterns for most genes and treatments.

**Observation 1 ***Let *Δ *be the pattern defined by the following outcomes: A *≺ *C, A ~ B, B ~ C. Then *Δ *is likely not a true pattern of response to the 3 treatments because the number of samples is insufficient.*

To see why this is true, given the above assumptions, suppose, to the contrary, that the number of samples are sufficiently large and the pattern Δ is a if such differences exist. Conversely, if no difference exists between two treatment groups, then they must be identical. Therefore, the outcomes *A ~ B *and *B ~ C *imply that *A ≡ B *and *B ≡ C*. But, this would imply that *A ≡ C*, which is a contradiction to the observed outcome *A *≺ *C*. Therefore, Δ is not true, and more samples are needed to determine the true pattern.

The same reasoning also shows that

**Observation 2 ***If a pattern contains *Δ, *then it is likely not a true pattern.*

**Figure 1:**
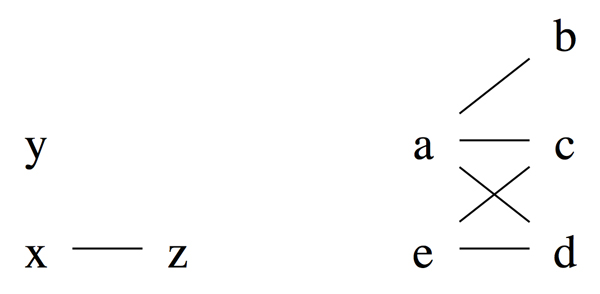
**(Left) Δ = {*x *≺ *z*, × ~ *y, y ~ z*}**. (Right) a pattern that is not true as it contains Δ (on elements *a, e*, and *b*).

As an illustration, Figure 1 shows Δ and a pattern that cannot be true as it contains Δ. To explore further properties of true patterns, we need a definition.

**Definition 1 ***A pattern P based on n elements t_1_, · · ·, t_n _is ***orderable ***if *∀*i, j such that t_i _~ t_j_, G(t_i_) = G(t_j_) and L(t_i_) = L(t_j_)*.

where *G*(*t_i_*) = {*t_k _|t_i _*≺ *t_k_*} is the set of elements "larger" than *t_i_*, and similarly *L*(*t_i_*) = {*t_k _|t_k _*≺ *t_i_*} is the set of elements "smaller" than *t_i_*.

**Observation 3 ***A pattern is orderable if and only if it does not contain *Δ.

To see this, suppose that a pattern *P *contains Δ = {*x *≺ *z*, × ~ *y, y ~ z}*. Then, since *x ~ y*, and *z *∈ *G*(*x*) but *z *∉ *G*(*y*), implies that *P *is not orderable. Conversely, if *P *does not contain Δ, then given any pair of *x *and *y *such that *x ~ y*, there cannot exist a *z *such that *x *≺ *z *and *y ~ z*. This implies *G*(*x*) = *G*(*y*). The same reasoning shows that *L*(*x*) = *L*(*y*). Thus, *P *is orderable. Observations 2 and 3 imply that

**Observation 4 ***True patterns are likely orderable.*

Although true patterns must be orderable, orderable patterns may not be true response of genes. If, however, already observed outcomes of types *A *≺ *B *are correct (with high probabilities), then additional samples do not change these and consequently the true pattern must be an *extension *of the observed pattern.

**Definition 2 ***Q is an ***orderable extension ***of P if (1) Q is orderable, and (2) *∀*i, j if P contains t_i _*≺ *t_j_, then Q also contains t_i _≺ t_j_*.

**Figure 2:**
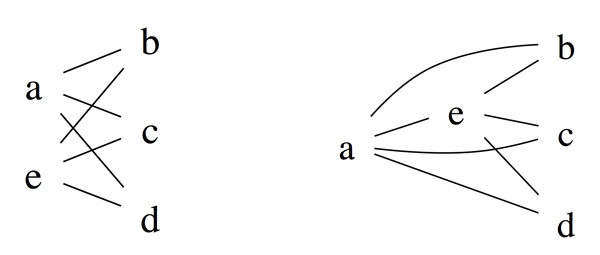
**(Left) an orderable pattern**. (Right) another orderable pattern. Both are *orderable extensions *of the pattern shown in Figure 1.

For instance, the patterns shown in Figure 2 are among the orderable extensions of the pattern shown in Figure 1. To predict true patterns, either we use large sample sizes and hope that observed patterns for most genes are orderable. This approach is expensive and there is no warranty that patterns of important genes of interest are orderable. Another approach to be described in the next section is to manipulate existing information based on already observed pattern and deduce most likely true patterns.

### Vary *α *to obtain orderable patterns

The confidence level *α *in comparison tests plays an important role in determining the outcomes (*A *≺ *B *or *B *≺ *A *or *A ~ B*) and ultimately whether or not the collective pattern is orderable. As an example, suppose that given 4 hypothetical treatments, the outcomes for 6 comparison tests are:

*• *p-value = 0.45 for the test if *µ_A _< µ_B_*.

*• *p-value = 0.03 for the test if *µ_A _< µ_C_*.

*• *p-value = 0.055 for the test if *µ_A _< µ_D_*.

*• *p-value = 0.03 for the test if *µ_B _< µ_C_*.

*• *p-value = 0.03 for the test if *µ_B _< µ_D_*.

*• *p-value = 0.45 for the test if *µ_C _< µ_D_*.

**Figure 3:**
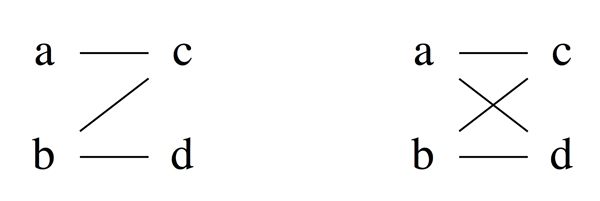
**Based on the same hypothetical expression data, (Left) observed pattern is not orderable with *α *= 0.05**. (Right) observed pattern is orderable with *α *= 0.06.

In independent statistical tests, *α *is fixed at a conventional small value such as 0.05 to reduce false positives [19]. If we set *α *at 0.05, then we will get the pattern {*A ~ B, A *≺ *C, A ~ D, B *≺ *C, B *≺ *D, C ~ D*}, which the left pattern in Figure 3. But this pattern is not orderable and cannot be true. If, however, we set *α *at a slightly higher value at 0.06, we will get an orderable pattern in Figure 3.

Since we have established that true patterns must be orderable, it makes sense to vary *α *conservatively in these dependent tests to achieve orderable patterns, which are more likely to be true patterns of gene response to treatments. Based on this idea, we can devise a procedure to determine an orderable pattern for a given gene, by finding a minimal *α *that yields a nontrivial orderable pattern. Algorithm 2 takes as input samples (replicates) of all treatment groups and finds an orderable pattern by increasing *α *(so long as it does not exceed a large value *α*max, e.g. 0.5) until a non-empty orderable patterns is obtained.

**Algorithm 2 **OrderablePattern(*S*)

1: Let *L *be the list of p-values computed in comparison tests for all treatment pairs based on samples *S*.

2: Sort *L *in increasing order (duplicates removed).

3: Let *m *be the largest index of *L *such that *L*[*m*] *< α*max

4: **for ***i *= 0 to *m ***do**

5:    *α ← L*[*i*]

6:    *P_α _*be the set of all outcomes computed based on *α*.

7:    **return ***P_α _*if it is orderable & non-empty

8: **return 0̸**

### Predict multiple patterns for each gene

A true pattern must be orderable, but an orderable might not be true. For example, either patterns in Figure 2 can be the true pattern depending on which *α *is used. When there are many patterns with approximately equal probabilities of being true, it makes more sense to predict multiple patterns for each gene. Further, because a gene might participate in multiple biological functions or molecular processes, assigning multiple patterns to a gene makes sense biologically.

To predict the most probable patterns for a given gene, we employ the bootstrap method [20,21] to resample (with replacement) from the set of gene expression replicates to create a large number of datasets for the purpose of approximating the sampling distribution of gene expression. Algorithm 3 computes the set of most probable patterns (and their probabilities) of a gene *g *using *M *iterations of bootstrap.

To be conservative in predicting multiple patterns, Algorithm 3 employs a heuristic which stipulates that if a pattern, *P*, is orderable based on a conventionally low *α *such as 0.05, then *P *is likely the true pattern. In this case, the algorithm returns *P *and its probability of 1. In case where *P *is not orderable at *α *= 0.05, we generate bootstrap samples for all treatments *ti*'s that might contribute to yielding orderable patterns. These are treatments involving in outcomes of the type *t_i _~ t_j _*in *P*. In each iteration *k*, bootstrap replicates are used to determine a pattern *P_k _*using either the fixed *α *(Algorithm 1) or variable *α *method (Algorithm 2). After a large number (*M*) of probabilistic bootstrap experiments, we can determine the set of patterns with their corresponding probabilities.

## Results

We aim to demonstrate the usefulness of varying *α *and assigning multiple patterns to genes using the proposed method. Specifically, we make comparisons in terms of counting orderable patterns (predicted to be true patterns) and measuring functional enrichment of gene sets. Additionally, we group genes with same patterns into clusters and compare functional enrichment of this technique against hierarchical clustering. Further, we analyze pharmacological activities of important genes (especially genes in enriched clusters identified by this method but missed by hierarchical clustering) known to be activated by the studied chemicals.

**Algorithm 3 **MultiplePatterns(*g*)

1: Let *S *= {*S*_1_*, · · ·, S_t_*} be the set of samples of gene *g*, where

*S_i _*is the set of samples of treatment *t_i_*.

2: *P ← Pattern*(*S*, 0.05) (Algorithm 1)

3: **if ***P *is orderable **then**

4:    **return **(*P*, 1)

5: Let *Q *be a multiset, initially empty.

6: **for ***k *= 1 to *M ***do**

7:    **for **each *i *such that ∃*j, t_i _~ t_j _*∈ *P ***do**

8:        replace samples *Si *with bootstrap replicates

9:    Determine pattern *P_k _*based on experimental and bootstrap replicates, using either Algorithm 1 or Algorithm 2.

10:    *Q *= *Q *∪ *P_k_*

11: **return **{(*P, f_P_*) | *P *∈ *Q, f_P _*is the frequency of *P *in *Q*}

### Design of experiments and evaluation

To validate our method, we used a gene expression data set came from a controlled study of samples of livers of Sprague-Dawley rats treated with either control diet or one of three chemopreventive compounds with well understood pharmacological activities, 5,6-benzoflavone (BNF), 3H-1,2-dithiole-3-thione (D3T) and 4-methyl-5-pyrazinyl-3H-1,2-dithiole-3-thione (OLT). This dataset had 5 samples in each of 4 treatment groups (including control group). It was available publicly with GEO accession number GSE8880 [6].

There totally 1737 significantly differentially expressed genes were selected using the Kruskal-Wallis procedure. Each gene is placed into a bin labelled by its pattern. When we assign multiple patterns (say *P*_1_*, · · ·, P_k_*) to a gene, we will place the gene to bins with labels *P*_1 _or *P*_2_, *· · · *, or *P_k_*. Thus, each bin contains genes with the same patterns.

Ultimately, the usefulness of a method lies in its ability to predict biological functions. To evaluate our method, we consider genes labelled with the same pattern as a gene cluster. We use DAVID to evaluate functional enrichment of each cluster. DAVID is a resource aimed at systematically extracting biological meaning from large gene lists. DAVID integrates biological information from most major public bioinformatics resources. We used the Gene Functional Classification tool of DAVID to extract highly-enriched clusters from each gene list. To quantify the degree of enrichment, we consider the number of functionally enriched clusters, number of enriched genes, and enrichment score that DAVID returns. All analyses were run with default parameters of DAVID.

### Varying *α *results in more orderable patterns

**Figure 4:**
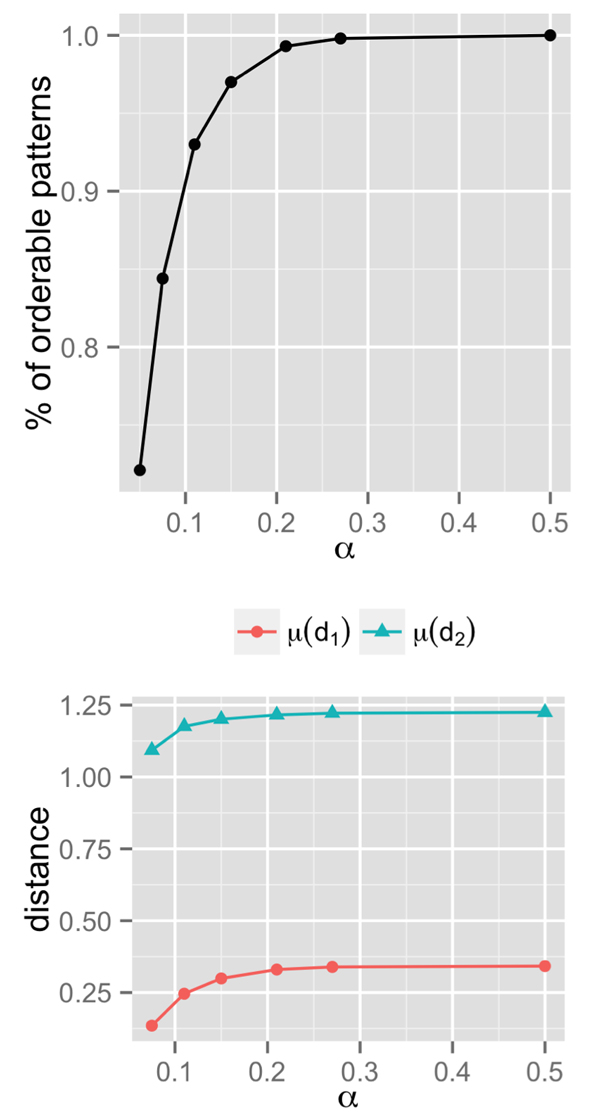
**(a) fraction of orderable patterns at increasing values of *α***. (b) structural difference between patterns acquired at *α *= 0.05 and at higher values.

With *α *fixed at 0.05, 1252 (72%) genes acquired 45 orderable patterns and 485 (28%) genes acquired 69 not orderable patterns. We observe that many orderable extensions can be obtained by modestly raising *α *beyond 0.05. Figure 4a shows that with *α ≤ *0.075, patterns of 84% of genes were orderable; and with *α ≤ *0.15, 97% of genes had orderable patterns. With *α ≤ *0.5, 100% of genes had 55 orderable patterns, including all 45 orderable patterns observed with *α *= 0.05 and 10 new patterns.

When the pattern *P *of a gene at *α *= 0.05 becomes another pattern *Q *at larger *α*, by construction, *Q *is an orderable extension of *P*. We will show quantitatively that the difference between *P *and *Q *is not very much. To compare the structural difference between patterns obtained at *α *fixed at 0.05 and their orderable extensions obtained by a higher *α*, we define the difference between two pattern *P *and *Q *as $d\left(P,Q\right)=\sum_{i=1}^{\left(\begin{array}{c} n\\ 2 \end{array}\right)}\delta \left(p_{i},\;q_{i}\right)$ where $\delta \left(p_{i},\;q_{i}\right)$ is 0 if the *i^th ^*outcome of *P *and *Q *is the same, and 1 if it is different. For example, if *P *and *Q *are the patterns shown in Figure 2, then *d*(*P, Q*) = 1 as they differ only in the comparison of *a *and *e*.

Figure 4b shows the average structural difference between a pattern observed with *α *fixed at 0.05 and its orderable extension observed varying *α*. This difference is denoted in the figure as *µ*(*d*_1_). As this number is well below 0.4 for all values of *α*, we see that on average a pattern is only very slightly different from its orderable extension. This number, however, does not tell the whole story, because 72% of genes acquired orderable patterns at *α *= 0.05 (meaning they are trivially their own orderable extensions). Thus, we proceed to analyze patterns that were not orderable at *α *= 0.05. The structural difference between these patterns and their orderable extensions is denoted in the figure as *µ*(*d*_2_). We see that this number is below 1.2 at all values of *α*. This means that on average adding roughly 1 outcome (of type *A *≺ *B*) to these patterns would make them orderable.

### Varying *α *results in better enrichment

We have shown that varying *α*, i.e. allowing different *α*'s for different genes, resulted in more orderable patterns. Since orderable patterns are more likely to be true patterns of gene response to treatments, it is expected that genes grouping in patterns that more likely true would result in more enrichment of the gene set. The enrichment of a gene list is described by DAVID's functional annotations in 3 aspects: (i) the number of enriched clusters (we refer to this in the figures as *E.cluster*), each of which is a subset of the gene list, (ii) the number of genes in each enriched clusters (*E.gene*), and (iii) the enrichment score of each cluster (*E.score*).

**Figure 5:**
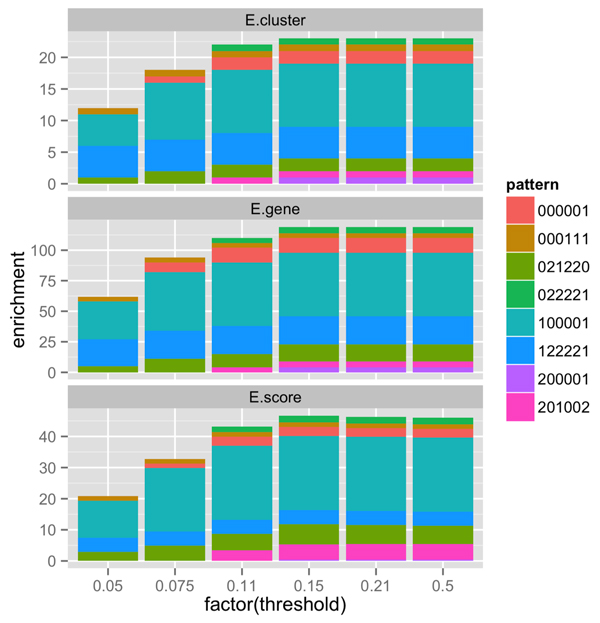
**Comparison of gene-set enrichment of clusters produced by fixing *α *at 0**.05 and allowing it to vary up to 0.5. The x-axis shows the maximum values of *α *that can be varied to obtain patterns. The y-axis shows the values of enrichment in terms of the number of enriched clusters (*E.cluster*), the number of genes in enriched clusters (*E.gene*), and the enrichment score (*E.score*). Each color represents a different pattern, codified by a 6-digit string.

Figure 5 shows that gene lists obtained from by varying *α *for each gene resulted in better enrichment than those obtained by fixing *α *at 0.05 for all genes. Specifically, with *α *fixed at 0.05, DAVID found 62 enriched genes in 12 enriched clusters, with a total enrichment score approximately 20.83. Meanwhile, for gene lists obtained with varying *α *up to 0.15 for each gene, DAVID founded 119 enriched genes in 23 enriched clusters with a total enrichment score of 46.70. Thus, gene lists produced by varying *α *up to 0.15 for each gene are twice as enriched as fixing *α *at 0.05.

Figure 5 shows that setting the maximum *α *larger than 0.15 did not improve enrichment very much. It seems that 0.15 is the optimal maximum threshold for this data set. For other data sets, it might be necessary to impose an upper bound on *α*, e.g. 0.15, to reduce false positives. This result suggests that doing so does not affect negatively functional enrichment very much.

An analysis of the results shown in Figure 5 reveals that varying *α *up to 0.15 yielded four *new *patterns (000001, 022221, 20001, and 201002) with a 26 enriched genes in 5 enriched clusters and total enrichment score approximately 10.37. These patterns were not observed for any genes when *α *was fixed at 0.05. Thus, varying *α *not only increased enrichment of existing patterns, it also discovers new enriched patterns.

### Assigning multiple patterns increases enrichment further

The motivation for assigning multiple patterns to a gene is that genes might have multiple biological functions or involve in multiple biological processes. If such is a case, we expect that such genes acquire several patterns with similarly high probabilities. We test this hypothesis by comparing gene-set enrichment of clusters produced by two different schemes: (i) one-pattern assignment scheme, which assigns only one pattern that is determined by real (experimental) replicates, and (ii) multiple-pattern assignment scheme, which assigns possibly multiple patterns, each of which is determined by replicates produced using bootstrap (as discussed in Methods).

In comparing these two assignment schemes, we chose to determine patterns by varying *α *up to 0.15, since the results have shown (as discussed in the previous section) that varying *α *with the upper bound of 0.15 resulted in most enrichment.

**Figure 6:**
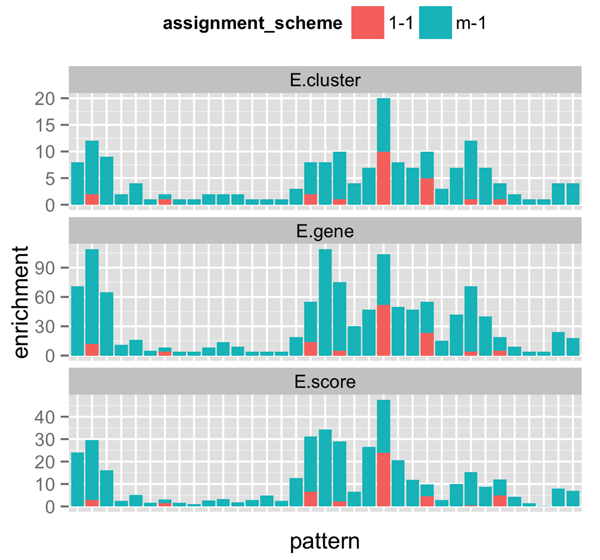
**Comparison of gene-set enrichment of clusters produced by one-pattern assignment (1-1) versus multiple-pattern assignment (m-1), with *α ≤ *0.15**. The x-axis lines up patterns with enriched clusters found by DAVID. The y-axis shows the values of enrichment in terms of the number of enriched clusters (*E.cluster*), the number of genes in enriched clusters (*E.gene*), and the enrichment score (*E.score*).

Figure 6 compares gene-set enrichment of the two assignment schemes. In the x-axis, we line up patterns with enriched clusters found by DAVID. Each bar corresponds to a pattern that contains enriched clusters. Patterns assigned to genes by the one-pattern assignment scheme are colored red. Patterns assigned to genes by the multiple-pattern assignment scheme are colored dark turquoise.

Figure 6 shows clearly that the multiple-pattern assignment scheme resulted in much more *and *much better enrichment than the one-pattern assignment scheme. Specifically, we observe that

*• *DAVID found enriched gene clusters in many more patterns assigned by multiple-pattern assignment scheme: 8 patterns by the one-pattern assignment scheme versus 35 patterns by the multiple-pattern assignment scheme. Further all of the 8 patterns were contained a subset of the 35 patterns. In other words, the multiple-pattern assignment scheme had *more enrichment *by discovering an additional 25 (3x more) more enriched patterns.

*• *An analysis of enriched patterns found in both schemes reveals that the enrichment for the multiple-pattern assignment scheme is much better in all 3 aspects: (i) the number of enriched clusters (*E.cluster*), (ii) the number of genes in enriched clusters (*E.gene*), and (iii) the enrichment scores for of enriched clusters (*E.score*). In other words, among patterns enriched by both schemes, the multiple-pattern assignment scheme had *better enrichment*.

This comparison shows much promise for the probabilistic approach to assigning multiple patterns to genes. It appears that resampling by bootstrap was helpful in determining true response patterns of genes.

### Clusters based on orderable patterns are better enriched

We may view the placement of genes into bins labelled by patterns as a clustering method because conceptually genes sharing the same patterns should share some biological functions. We shall analyze the predictive power of our method as a clustering method and compare the enrichment of gene clusters produced by this method and gene clusters produced by hierarchical clustering. Although there are better clustering methods for specific scenarios, hierarchical clustering remains popular as a general-purpose clustering technique and as such it is a useful gold standard for comparison. We used Pearson correlation as the distance between two gene expression vectors; each gene expression vector consists of mean expressions of all treatment groups of a gene. Average linkage was used as a measure of similarity between two clusters.

For comparison, it makes more sense to compare hierarchical clustering to our deterministic one-pattern assignment scheme, which assigns only one pattern to each gene, although the probabilistic, multiple-pattern assignment yielded much more enrichment in all aspects than the deterministic one-pattern assignment did. This comparison is more appropriate because hierarchical clustering assigns each gene to only one cluster and in essence is similar to the deterministic onepattern assignment scheme.

For an appropriate comparison, we chose the hierarchy of clusters (for hierarchical clustering) that gave exactly 78 clusters. Since each hierarchy gives a different number of clusters, one must selected for comparison. And in our approach, an *α *threshold results in a specific number of clusters for set of differentially expressed genes. Specifically, if we vary *α *to maximally 0.15 (*α*_max _= 0.15), we get exactly 78 clusters for our dataset. Since we chose this threshold for comparison, we needed to chose an appropriate hierarchy for hierarchical clustering to give the same number of clusters.

As shown in Table 1 DAVID analyzed the set of clusters produced by hierarchical clustering and found 8 clusters that contained a total of 11 enriched clusters (denoted *E.cluster*). One cluster contained 3 enriched clusters; another contained 2 enriched clusters; and each of the other contained 1 enriched cluster. These enriched clusters contained between 4 and 16 genes (denoted *E.genes*). The enrichment score (*E.score*) for each enriched cluster was between 0.58 and 5.82.

**Table 1:** Functional enrichment of clusters produced by hierarchical clustering (hc) and our method for variable *α ≤ *0.15.

method	cluster-id	E.cluster	E.gene	E.score
hc	c70	3	16	5.8176
hc	c35	2	8	3.8396
hc	c11	1	4	3.0557
hc	c72	1	4	2.6481
hc	c34	1	4	1.2998
hc	c71	1	4	1.0265
hc	c26	1	4	0.8574
hc	c77	1	4	0.5839
*α ≤ *0.15	100001	10	52	23.8291
*α ≤ *0.15	021220	2	14	6.4583
*α ≤ *0.15	201002	1	5	4.9576
*α ≤ *0.15	122221	5	23	4.5289
*α ≤ *0.15	000001	2	12	2.8358
*α ≤ *0.15	022221	1	5	2.1743
*α ≤ *0.15	000111	1	4	1.5216
*α ≤ ***0.15**	**200001**	**1**	**4**	**0.4007**

Very interestingly, DAVID also found 8 clusters in the set of clusters produced by our method. These 8 clusters, however, contained a total of 23 enriched clusters; this is more than double the number of enriched clusters for hierarchical clustering. These 23 enriched clusters contained a total of 119 enriched genes; this is also more than double the number of enriched genes for hierarchical clustering. The individual and overall enrichment scores of enriched clusters in our method are again more than twice those in hierarchical clustering. In summary, our method with *α ≤ *0.15 produced clusters that are more than twice as enriched as those produced by hierarchical clustering.

An important distinction between clustering by comparison-based patterns and by hierarchical clustering is that comparison-based patterns clusters are labelled, while clusters produced by hierarchical clustering (or any other *unsupervised *method) are unlabeled. The labels of comparison-based clusters are patterns that specify precisely how genes placed in such clusters respond to all treatment pairs. Such labelled can be meaningful annotations and carry useful meanings for subsequent analyses. By contrast, for hierarchical clustering, other than the fact that genes in the same clusters are similar based on Pearson correlation (some other distance metric), it is harder to interpret genes that are in the same clusters.

A closer analysis of genes in enriched clusters produced by the two methods shows that 28 out of 48 genes in enriched clusters produced by hierarchical clustering were also in enriched clustering found by our method. Hierarchical clustering discovered 20 genes in enriched clusters that our method did not. On the other hand, our method discover 99 genes in enriched clusters that hierarchical clustering did not.

### Biological associations of genes in discovered patterns are confirmed by literature

**Figure 7:**
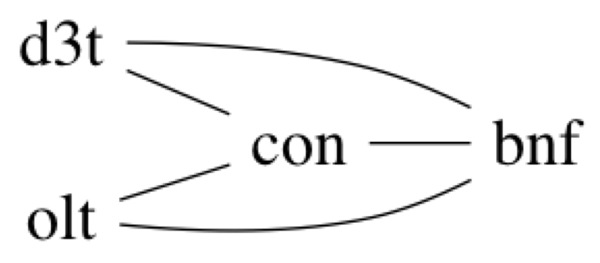
**Pattern contains 4 transcription factors *Nfe2l2, Klf2, Egr1*, and *Irf8 *in an enriched cluster found by DAVID**. These genes participate in multiple biological networks.

Additional analyses of genes in enriched clusters discovered by our method and missed by hierarchical clustering showed that some of these genes were in fact associated in multiple biological networks and had interesting known pharmacological behaviors. The pattern shown in Figure 7 includes 62 genes, among which 4 transcription factor-encoding genes (*Nfe2l2, Klf2, Egr1*, and *Irf8*) belong to an enriched cluster found by DAVID. These 4 transcription factors were grouped together in one cluster by our method, but were placed in different clusters by hierarchical clustering.

**Figure 8:**
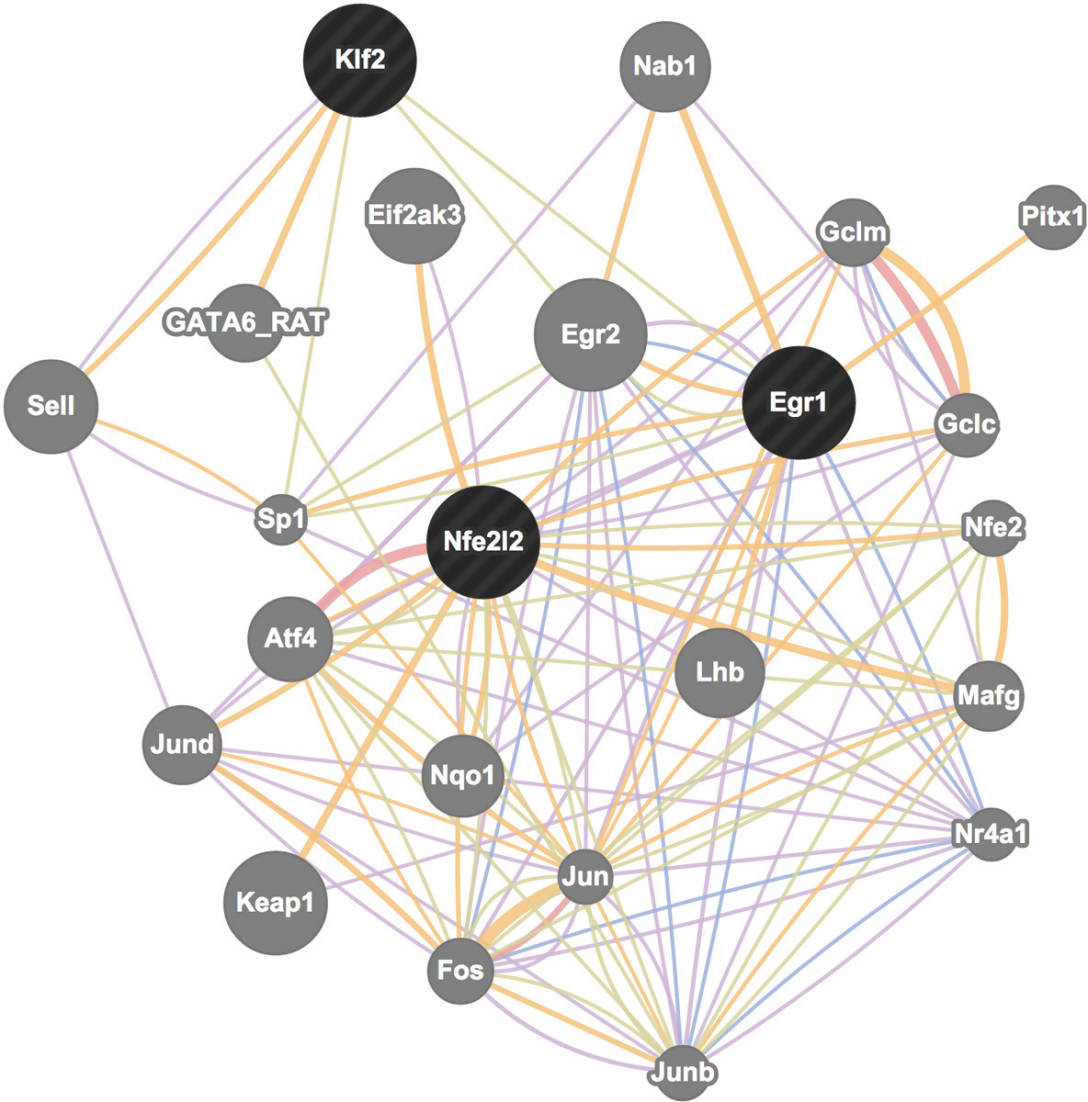
**Biological networks that include *Nfe2l2, Klf2, Egr1*, and *Irf8***. Edge colors encode different types of networks. Related genes in the networks are in grey circles.

Three of the four transcription factors (*Nfe2l2, Klf2*, and *Egr1*) have been known to participate in five biological networks (Figure 8): (i) co-expression network (violet edges) supported by 20 publications; (ii) predicted functional network (yellow edges) supported by 13 publications; (iii) protein-protein interaction network (pink edges) supported by 5 data sources, (iv) co-localization network (blue edges) supported by 1 publication, and (v) shared protein domain network (olive edges) supported 2 data sources. These networks were obtained by GeneMANIA [22] based on the current literature and many different sources of functional association data. An interesting finding from these networks is that *Egr1, Klf2*, and two other genes *Sp1 *and *Egr2 *mutually share protein domains.

Among these transcription factors, *Nfe2l2 *is known to play an important role in many reported pharmacological activities by the studied chemo-prenventive chemical compounds (BNF, D3T, and OLT). *Nfe2l2 *is a master regulator of the expression of antioxidant response element-dependent genes, which produce proteins responsible for the detoxication of electrophiles and reactive oxygen species [23,24]. Induction of studied chemical treatments BNF, D3T, and OLT on *Nfe2l2 *were revealed in many studies. Dewa et al. [25] revealed that BNF induced many *Nfe2l2*-regulated genes in a study of oxidative stress responses in the livers of rats. Kobayashi et al. [26] showed that *Nfe2l2 *(together with Keap1) was activated in response to D3T treatment in a study on Zebrafish. Tran et al. [7] also confirmed that many *Nfe2l2*-dependent genes, particularly detoxifying and antioxidant proteins, respond to D3T and OLT treatments in a study to examine pharmacological structure-activity relationships of these compounds in rat livers. Dong et al. [27] demonstrated that D3T can induce *Nrf2 *activation and prevent ethanol-induced oxidative stress and apoptosis in a study on PC12 cells. The results of these studies support the hypothesis that *Nfe2l2 *plays an important role in pharmacological activities by the studied chemical treatments BNF, D3T, and OLT.

## Conclusions

We introduce a novel method for the analysis of gene expression patterns in studies involving multiple treatments. We derived crucial properties that *orderable *comparison-based patterns are more likely to be true patterns. This property helps exploit the interdependencies among statistical tests whose outcomes constitute observed patterns. Consequently, we are able to increase *α*, the threshold used in the statistical tests, beyond the traditional value of 0.05 to enable a more accurate prediction of true patterns. Objective analyses by DAVID confirmed that increasing *α *carefully in this way indeed yielded more functional enrichment.

Another novel aspect of this method is in the probabilistic assignment of multiple patterns to each gene. While fuzzy clustering of genes have been introduced, multiple pattern assignment based on bootstrap resampling is a refreshing direction. We further showed that the multiple-pattern assignment scheme increased functional enrichment manyfold. By assigning multiple patterns to a gene, the method discovered new enriched patterns and at the same time increased the enrichment of already discovered enriched clusters.

A comparison to hierarchical clustering shows that the one-pattern assignment scheme produced the same number of enriched clusters, but those clusters were twice as enriched in all three different aspects: the number of enriched groups in each cluster, the number of genes in each enriched group, and the total enrichment score of each group. We compared hierarchical clustering to the one-pattern assignment scheme because both methods place a gene into one cluster, although the multiple-pattern assignment scheme is much better than the one-pattern assignment scheme in terms of producing functionally enriched gene sets. More thorough analysis of three transcription factors grouped together in an enriched cluster found by our method and missed by hierarchical clustering showed that these transcription factors participated in multiple biological networks. One particular transcription factor, *Nfe2l2*, was known to play an important role in many pharmacological activities related to the studied chemo-preventive chemical compounds.

This method is particularly useful in studies with many drugs, with small sample sizes or contain highly variantly expressed genes. If the sample size is sufficient but still small enough that conventional methods might suffer to gauge true response patterns, our approach might be beneficial in its exploitation of the dependencies among interdependent statistical tests. Specifically, the exploitation of orderable patterns will help vary *α *in an intelligent manner to predict true order of gene response to treatments accurately.

## Competing interests

The authors declare that they have no competing interests.

## Authors' contributions

Nam S. Vo (NSV) implemented the methods, developed software and scripts for analyses; performed simulations and experiments; helped analyze results. Vinhthuy Phan (VP) developed the theory, proofs; designed the experiments and simulations.

## Declarations

Publication charges for this work were funded by NSF grant CCF-1320297 to VP.

This article has been published as part of *BMC Bioinformatics *Volume 15 Supplement 11, 2014: Proceedings of the 11th Annual MCBIOS Conference. The full contents of the supplement are available online at http://www.biomedcentral.com/bmcbioinformatics/supplements/15/S11.
